# Supplementary material for: Correlations among the nursing work environment, traumatic stress, and professional quality of life in Chinese midwives: A cross-sectional study
Source: PLoS One. 2025 Oct 8;20(10):e0328686. doi: 10.1371/journal.pone.0328686 (PMC12507232; doi:10.1371/journal.pone.0328686)
Supplement: Attachment — Submitted filename: PONE- Dr.Rasha Ibrahim.docx [file pone.0328686.s002.docx]

PLOS ONE

**The Correlation Between Nursing Work Environment, Traumatic Stress and Compassion Fatigue among Chinese Midwives working in the delivery room: A Cross- Sectional Study**

--Manuscript Draft--

| **Manuscript Number:** | PONE-D-24-58466 |
| --- | --- |
| **Article Type:** | Research Article |
| **Full Title:** | The Correlation Between Nursing Work Environment, Traumatic Stress and Compassion Fatigue among Chinese Midwives working in the delivery room: A Cross- Sectional Study |
| **Short Title:** | The Correlation Between Nursing Work Environment, Traumatic Stress and Compassion Fatigue |
| **Corresponding Author:** | lixia qu  Zhengzhou University zhengzhou, CHINA |
| **Keywords:** | Introduction: Compassion fatigue poses a significant threat to healthcare workers working in high-intensity, high-risk, and high-stress environments. Limited research has been conducted on the nursing work environment, traumatic stress, and their role in c |
| **Abstract:** | Introduction : Compassion fatigue poses a significant threat to healthcare workers working in high-intensity, high-risk, and high-stress environments. Limited research has been conducted on the nursing work environment, traumatic stress, and their role in compassion fatigue among midwives. The study aimed to identify the correlation between the nursing working environment, traumatic stress, and compassion fatigue among Chinese midwives. We also aimed to identify factors related to participants' compassion satisfaction, burnout, and secondary traumatic stress.  Methods: An online questionnaire was administered to 232 midwives working in the delivery room from 59 hospitals in Henan Province, China. Participants were recruited through convenience sampling from November to December 2023. Data collection tools were: Demographic and professional characteristics Questionnaire, Traumatic Stress Scale for Midwives (consists of frequency and impact), Nursing Work Environment Scale, Professional Quality of Life Scale to measure compassion fatigue (concludes burnout and secondary traumatic stress) and compassion satisfaction.  Descriptive statistics, Mann-Whitney U test, Kruskal-Wallis H test, Spearman relationship, and multiple linear regression were employed for data analysis.  Results: The majority reported a moderate level of compassion  satisfaction (35.18±7.703) and burnout (25.33±4.334) and a low level of secondary traumatic stress (21.50±5.464). Nursing work environment, the impact and the frequency of traumatic stress could predict 51.4%, 25.4%, and 12.6% of compassion satisfaction. The nursing work environment and the impact of traumatic stress could explain 19.9% and 39.0% of the variance in burnout. The nursing work environment and the impact of traumatic stress could account for 17.2% and 33.8% of secondary traumatic stress.  Conclusion: The associations we identified between the nursing work environment, traumatic stress, and compassion fatigue suggest the potential importance of implementing nursing work environment and developing strategies such as trauma- informed care education and trauma management. These strategies could help reduce midwives' compassion fatigue, thereby promoting their health and well-being. |
| **Order of Authors:** | xiaofei zhang |
|  | bing lun |
|  | haojie ge |
|  | lixia qu |
| **Additional Information:** |  |
| **Question** | **Response** |

| **Financial Disclosure**  Enter a financial disclosure statement that describes the sources of funding for the work included in this submission. Review the [submission guidelines](http://journals.plos.org/plosone/s/submission-guidelines#loc-financial-disclosure-statement) for detailed requirements. View published research articles from [*PLOS ONE*](https://journals.plos.org/plosone/) for specific examples.  This statement is required for submission and **will appear in the published article** if the submission is accepted. Please make sure it is accurate. | Yes |
| --- | --- |
| **Funded studies**  Enter a statement with the following details:   - Initials of the authors who received each award - Grant numbers awarded to each author - The full name of each funder - URL of each funder website - Did the sponsors or funders play any role in the study design, data collection and analysis, decision to publish, or preparation of the manuscript? |  |
| Did you receive funding for this work? |  |
| Please add funding details.  as follow-up to "**Financial Disclosure**  Enter a financial disclosure statement that describes the sources of funding for the work included in this submission. Review the [submission guidelines](http://journals.plos.org/plosone/s/submission-guidelines#loc-financial-disclosure-statement) for detailed requirements. View published research articles from [*PLOS ONE*](https://journals.plos.org/plosone/) for specific examples.  This statement is required for submission and **will appear in the published article** if the submission is accepted. Please make sure it is accurate. | Henan Provincial Health Commission |

| **Funded studies**  Enter a statement with the following details:   - Initials of the authors who received each award - Grant numbers awarded to each author - The full name of each funder - URL of each funder website - Did the sponsors or funders play any role in the study design, data collection and analysis, decision to publish, or preparation of the manuscript? |  |
| --- | --- |
| Did you receive funding for this work?" |  |
| Please select the country of your main research funder (please select carefully as in some cases this is used in fee calculation).  as follow-up to "**Financial Disclosure**  Enter a financial disclosure statement that describes the sources of funding for the work included in this submission. Review the [submission guidelines](http://journals.plos.org/plosone/s/submission-guidelines#loc-financial-disclosure-statement) for detailed requirements. View published research articles from [*PLOS ONE*](https://journals.plos.org/plosone/) for specific examples.  This statement is required for submission and **will appear in the published article** if the submission is accepted. Please make sure it is accurate. | CHINA - CN |
| **Funded studies**  Enter a statement with the following details:   - Initials of the authors who received each award - Grant numbers awarded to each author - The full name of each funder - URL of each funder website - Did the sponsors or funders play any role in the study design, data collection and analysis, decision to publish, or preparation of the manuscript? |  |
| Did you receive funding for this work?" |  |
| **Competing Interests** | The authors have declared that no competing interests exist. |

| Use the instructions below to enter a competing interest statement for this submission. On behalf of all authors, disclose any [competing interests](http://journals.plos.org/plosone/s/competing-interests) that could be perceived to bias this work—acknowledging all financial support and any other relevant financial or non- financial competing interests.  This statement is required for submission and **will appear in the published article** if the submission is accepted. Please make sure it is accurate and that any funding sources listed in your Funding Information later in the submission form are also declared in your Financial Disclosure statement.  View published research articles from  [*PLOS ONE*](http://journals.plos.org/plosone/) for specific examples. |  |
| --- | --- |
| **NO authors have competing interests**  Enter: *The authors have declared that no competing interests exist.*  **Authors with competing interests**  Enter competing interest details beginning with this statement:  *I have read the journal's policy and the authors of this manuscript have the following competing interests: [insert competing interests here]* |  |
| * typeset |  |
| **Ethics Statement**  Enter an ethics statement for this submission. This statement is required if the study involved:   - Human participants - Human specimens or tissue - Vertebrate animals or cephalopods - Vertebrate embryos or tissues | The department of Ethics Committee, Zhengzhou University provided ethical approval (No: ZZUIBRB2020-52). |

| - Field research   Write "N/A" if the submission does not require an ethics statement.  General guidance is provided below. Consult the [submission guidelines](http://journals.plos.org/plosone/s/submission-guidelines#loc-guidelines-for-specific-study-types) for detailed instructions. **Make sure that all information entered here is included in the Methods section of the manuscript.** |  |
| --- | --- |

| **Format for specific study types**  **Human Subject Research (involving human participants and/or tissue)**   - Give the name of the institutional review board or ethics committee that approved the study - Include the approval number and/or a statement indicating approval of this research - Indicate the form of consent obtained (written/oral) or the reason that consent was not obtained (e.g. the data were analyzed anonymously)   **Animal Research (involving vertebrate animals, embryos or tissues)**   - Provide the name of the Institutional Animal Care and Use Committee (IACUC) or other relevant ethics board that reviewed the study protocol, and indicate whether they approved this research or granted a formal waiver of ethical approval - Include an approval number if one was obtained - If the study involved *non-human primates*, add *additional details* about animal welfare and steps taken to ameliorate suffering - If anesthesia, euthanasia, or any kind of animal sacrifice is part of the study, include briefly which substances and/or methods were applied   **Field Research**  Include the following details if this study involves the collection of plant, animal, or other materials from a natural setting:   - Field permit number - Name of the institution or relevant body that granted permission |  |
| --- | --- |
|  |  |
| **Data Availability**  Authors are required to make all data underlying the findings described fully available, without restriction, and from the time of publication. PLOS allows rare exceptions to address legal and ethical concerns. See the [PLOS Data Policy](http://journals.plos.org/plosone/s/data-availability) and [FAQ](http://journals.plos.org/plosone/s/data-availability#loc-faqs-for-data-policy) for detailed information. | Yes - all data are fully available without restriction |

| A Data Availability Statement describing where the data can be found is required at submission. Your answers to this question constitute the Data Availability Statement and **will be published in the article**, if accepted. |  |
| --- | --- |
| **Important:** Stating ‘data available on request from the author’ is not sufficient. If your data are only available upon request, select ‘No’ for the first question and explain your exceptional situation in the text box. |  |
| Do the authors confirm that all data underlying the findings described in their manuscript are fully available without restriction? |  |
| **Describe where the data may be found in full sentences. If you are copying our sample text, replace any instances of XXX with the appropriate details.**   - If the data are **held or will be held in a public repository**, include URLs, accession numbers or DOIs. If this information will only be available after acceptance, indicate this by ticking the box below. For example: *All XXX files are available from the XXX database (accession number(s) XXX, XXX.*). - If the data are all contained **within the manuscript and/or Supporting Information files**, enter the following: *All relevant data are within the manuscript and its Supporting Information files.* - If neither of these applies but you are able to provide **details of access elsewhere**, with or without limitations, please do so. For example:   *Data cannot be shared publicly because of [XXX]. Data are available from the XXX Institutional Data Access / Ethics Committee (contact via XXX) for researchers who meet the criteria for access to confidential data.*  *The data underlying the results presented in the study are available from (include the name of the third party* | The questionnaire collection platform (Wenjuanxing) in China is used to collect and store the data on a password controlled computer, which can only be accessed by the author of this study |

| *and contact information or URL).*   - This text is appropriate if the data are owned by a third party and authors do not have permission to share the data.   * typeset |  |
| --- | --- |
| Additional data availability information: | Tick here if your circumstances are not covered by the questions above and you need the journal’s help to make your data available. |

Manuscript	[Click here to access/download;Manuscript;renamed_e5114-](https://www2.cloud.editorialmanager.com/pone/download.aspx?id=38669208&guid=dcc2b82a-996d-41b6-a6bd-227ed022e0b9&scheme=1)[<https://www2.cloud.editorialmanager.com/pone/download.aspx?id=38669208&guid=dcc2b82a-996d-41b6-a6bd-227ed022e0b9&scheme=1> 1.doc](https://www2.cloud.editorialmanager.com/pone/download.aspx?id=38669208&guid=dcc2b82a-996d-41b6-a6bd-227ed022e0b9&scheme=1)

**The correlation between nursing work environment, traumatic stress and compassion fatigue among Chinese midwives: A cross-sectional study**

Xiaofei Zhang, undergraduate^a^ Bing Lun, BSN, Nurse-in-charge^b^ Haojie Ge, undergraduate^a^

Lixia Qu, MSN, Senior Lecturer ^a^

**a** School of Nursing and Health, Zhengzhou University, Zhengzhou, 475000, China

**b** delivery room, Maternal and Child Health Hospital of Henan Province, Zhengzhou, 475000, China

Xiaofei Zhang	Email: [15981863915@163.com](mailto:15981863915@163.com) Bing Lun	Email: [lunbing1985@163.com](mailto:lunbing1985@163.com)

Haojie Ge	Email: [piggy0413000@gmail.com](mailto:piggy0413000@gmail.com) Lixia Qu	Email: [qulixia@zzu.edu.cn](mailto:qulixia@zzu.edu.cn)

# Corresponding author

Email address: [qulixia@zzu.edu.cn](mailto:qulixia@zzu.edu.cn) Postal Code: 475000

Tel: +86 18336356708

# Word count of the text: 3605

(1) **Conflict of Interest**

The authors have no conflicts of interest.

# (2) Acknowledgments

The authors would like to gracefully thank all the midwives who participated in this study.

# The Correlation Between Nursing Work Environment, Traumatic Stress and Compassion Fatigue among Chinese Midwives working in the delivery room: A Cross-Sectional Study

**Abstract**

**Introduction:** Compassion fatigue poses a significant threat to healthcare workers working in high-intensity, high-risk, and high-stress environments. Limited research has been conducted on the nursing work environment, traumatic stress, and their role in compassion fatigue among midwives. The study aimed to identify the correlation between the nursing working environment, traumatic stress, and compassion fatigue among Chinese midwives. We also aimed to identify factors related to participants' compassion satisfaction, burnout, and secondary traumatic stress.

**Methods:** An online questionnaire was administered to 232 midwives working in the delivery room from 59 hospitals in Henan Province, China. Participants were recruited through convenience sampling from November to December 2023. Data collection tools were: Demographic and professional characteristics Questionnaire, Traumatic Stress Scale for Midwives (consists of frequency and impact), Nursing Work Environment Scale, Professional Quality of Life Scale to measure compassion fatigue (concludes burnout and secondary traumatic stress) and compassion satisfaction. Descriptive statistics, Mann-Whitney U test, Kruskal-Wallis H test, Spearman relationship, and multiple linear regression were employed for data analysis.

**Results:** The majority reported a moderate level of compassion satisfaction (35.18±7.703) and burnout (25.33±4.334) and a low level of secondary traumatic stress (21.50±5.464). Nursing work environment, the impact and the frequency of traumatic stress could predict 51.4%, 25.4%, and 12.6% of compassion satisfaction. The nursing work environment and the impact of traumatic stress could explain 19.9% and 39.0% of the variance in burnout. The nursing work environment and the impact of traumatic stress could account for 17.2% and 33.8% of secondary traumatic stress.

**Conclusion:** The associations we identified between the nursing work environment, traumatic stress, and compassion fatigue suggest the potential importance of implementing

nursing work environment and developing strategies such as trauma-informed care education and trauma management. These strategies could help reduce midwives' compassion fatigue, thereby promoting their health and well-being.

**Keywords:** compassion fatigue, compassion satisfaction, nursing work environment, traumatic stress, midwives

# Introduction

The mental and psychological health of health care workers is very important for the organization. Compassion fatigue (CF) is a risk faced by health care workers working in hospitals due to the daily rhythms, expectations, patient burden, and acuity that workers face and traumatic events.^1,2^ Compassion fatigue is "the emotional, moral and physical distress, which occurs as a consequence of caring and bearing witness to the suffering of others".^3^Midwives, as professional and responsible persons, with the cooperation of childbearing mothers, care for and support them as they transition into mothers. Although birth is often a joyful event, some women may experience unpredictable complications during childbirth called traumatic events,^4^ which is also traumatic for midwives and can trigger strong emotional responses,^5^ make midwives painful. Moreover, the intimate relationship between midwives and childbearing mothers is a decisive core feature of midwife work,^6^ which puts them at high risk of compassion fatigue when witnessing traumatic childbirth, leading to negative emotional outcomes, like distress, exhaustion, depression, frustration, absenteeism, and so on,^7^ these negative feelings prevent development and maintenance of caring relationship with childbearing mothers and affect the care they provide.

Compassion fatigue can have a negative impact on health care workers, patients, and

hospitals.^8^ Health care workers suffering from compassion fatigue may experience symptoms of mental and physical exhaustion, leading to challenges in the ability to provide compassionate care for patients,^5^ and compassion fatigue can negatively impact patient safety and satisfaction,^9^ which also contribute to missed days of work and higher attrition rates among health care workers,^10^ negatively impacting hospital organizations.

Compassion fatigue consists of burnout and secondary traumatic stress. "Burnout is associated with feelings of hopelessness and difficulties in dealing with work or in doing your job effectively."^11^It can have a series of negative impacts on patients, medical institutions, the nursing profession, and the individual physical and mental health of nurses.^12^ Secondary traumatic stress is defined as "work-related, secondary exposure to people who have experienced extremely or traumatically stressful events."^11^It is characterized by insomnia, fears, nightmares, and avoidance behavior.^13^ The pressure caused by such trauma increases malpractice, leads to loss of motivation, and reduces compassion in the nursing process.^14^

Besides, health care workers can also derive pleasure from helping patients, such pleasure is called compassion satisfaction.^11^ Compassion fatigue and compassion satisfaction collectively define the professional quality of life,^11^ representing both negative and positive aspects of the quality of professional life.

Nursing work environment refers to the organizational characteristics of the workplace that promote or limit professional nursing practice.^15^ Research has shown a strong correlation between the nursing work environment and nurses' well-being, outcome of patients, and quality of care.^16,17^ A healthier work environment means better job satisfaction, collaboration, support, and a psychological sense of belonging.^17,18^ Through Working in a healthier work environment, health care workers are less likely to resign and turnover^19^ and can make the greatest positive contribution to patients and organizations.^20^ Previous research indicated that negative emotions may be exacerbated because of work pressure and unsupportive work environment,^21^ and distress about work environment was an important determinant of higher compassion fatigue.^22^ Evidence has demonstrated that overburdened work environments may make nurses more susceptible to compassion fatigue.^23^ However, certain work environment characteristics may reduce compassion fatigue. For example, a supportive team environment, better professional development, and more professional autonomy can mitigate compassion fatigue,^24,25^ besides, meaningful recognition and acknowledgment of individual contributions can regard as a motivational factor, reducing compassion fatigue, and promoting compassion satisfaction.^26^

Reducing compassion fatigue among midwives is essential for their well-being and the

outcomes for childbearing mothers and the organization. In China, midwifery human resources are in a state of scarcity.^27^ Moreover, the delivery room is a high-risk, high-intensity, and high-stress environment, midwives need to face and resolve many birth-related situations independently, traumatic stress brought by complicated delivery makes them face emotional challenges such as psychological distress and compassion fatigue. However, little is known about the correlation between the nursing work environment, traumatic stress, and compassion fatigue among Chinese midwives who working in the delivery room. On August 20, 2021, the "three children" birth policy was officially implemented,^28^ significantly increasing the proportion of elderly pregnant and postpartum women and posing greater

challenges to midwives. This study aimed to explore the relationship between nursing work environment, traumatic stress, and compassion fatigue to develop effective measures to improve outcomes for childbearing mothers, healthcare organizations, and midwives' health and well-being.

# Methods Study design

A cross-sectional survey of midwives who working in the delivery room was conducted.

# Setting and participants

Data were collected from a voluntary convenience sample of midwives from 59 hospitals in Henan Province, China, from November to December 2023. The inclusion criteria were: 1) as a midwife with at least one year of experience in the delivery room, 2) as registered nurses who also hold midwifery endorsements. The information of all participants is strictly confidential, and the electronic information was stored in a locked computer, and was seen only by researchers.

G*power 3.1 software was adopted for sample size calculation.^30^ The parameters set included an effect size of d=0.15, an alpha level of 0.05, and a power level of 0.95. Results showed a minimum sample size of 213. Our sample size met the requirement.

# Data collection process

Before the survey, we recruited delivery room head nurses as investigators in the Henan Province delivery room head nurse group (established at a meeting), with one head nurse from each hospital. Finally, 71 head nurses agreed to participate and trained uniformly. The trained investigators informed the midwives of this study's purpose and the voluntary nature of participation, consisting the right to withdraw at any time and the right to anonymity, and they used in a unified guiding language. The questionnaire star link (an online survey tool) was sent via WeChat to the midwives who met the inclusion criteria. To prevent duplicate submissions, a single IP can only be filled out once, and each option is set as a mandatory option. All information collected from research participants is protected and only accessible to the research team.

# Ethical considerations

The department of Ethics Committee, Zhengzhou University provided ethical approval

(No: ZZUIBRB2020-52). This study was designed and reported in alignment with the Strengthening the Reporting of Observational Studies in Epidemiology (STROBE) guidelines.^29^ In regarding to informed consent, the first question designed in the questionnaire is whether you agree to participate in this study, the answer is yes or no. If you choose no, you cannot continue to answer the question, If you choose yes, you can continue to answer. The research report did not disclose the names of the participants, and all information collected from them is protected and only accessible to the research team.

# Measurement

Demographic and professional characteristics questionnaire is a self-report tool, including age, marital status, educational level, hospital level, professional title, employment form, duty, years as a midwife, major shifts, frequency of night shift per month, personal monthly income, weekly working time, number of deliveries per week on average, sleep quality and health condition.

Nursing Work Environment Scale (NWES) is a 26-item, self-report tool developed by Ye,^31^ which comprising 7 subscales: career development, leadership and management, doctors and nurses relationship, recognition atmosphere, professional autonomy, basic guarantees, and sufficient human resources. Each item is presented on a 6-point Likert scale, ranging from 1 (strongly disagree) to 6 (strongly agree). Results range from 26 to 156, a higher score shows a better nursing work environment for the participant. The Cronbach's α was 0.946.^31^ Permission to use this tool was obtained.

ProQol was assessed using the Professional Quality of Life Scale,^11^ and we used the Chinese version.^32^ This is a self-report questionnaire concluding 30 items, each item is rated on a 5-point Likert scale of 1="never" to 5="very often". The scale consists of three subscales: compassion satisfaction (CS), burnout (BO), and second traumatic stress (STS), each subscale has 10 items. Items 1,4,15,17, and 29 of the Likert scale are reverse-scored. Scores on the subscale can range from 10 to 50, with a higher score signifying a higher lever of regard. According to Stamm (2010), scores can be categorised as low (≦22), moderate

(23-41) and high (≧42) in each of the three subscales. The ProQoL has shown good

reliability, Cronbach's α=0.87, 0.72 and 0.80, respectively, for CS, BO, and STS.^33^ Chinese version also demonstrated good internal consistency.^32^

Traumatic Stress Scale for Midwives (TSSM)^34^ was employed to measure traumatic stress. The Chinese version, translated and revised by Pu^35^, was used for this study. The scale has 2 dimensions, including frequency and impact caused by traumatic stress. Each subscale includes 15 identical items. The two subscales are presented on a 4-point likert scale. The frequency subscale ranges from 0 (never) to 3 (always), the impact subscale ranges from 0 (not at all) to 3 (very), and the total score for each subscale is 0-45. A higher frequency score indicates more traumatic stress experience and a higher impact score indicates more hurt from traumatic stress experience, the Cronbach's alpha was 0.832 and 0.962 in our study.

# Data analysis

IBM SPSS 26.0 was employed to analysis the data. Q-Q test was adopted to analyze whether the data was aligned with normal distribution. Due to all measurement data not meeting normal distribution, the Mann-Whitney U and Kruskal-Wallis H tests were employed to analyze participants' compassion satisfaction, burnout, and secondary traumatic stress associated with demographic and professional characteristics, and a statistically significant difference was indicated by *P*<0.10.^36^ Spearman correlation analysis was adopted to analyze the correlations among participants' traumatic stress, nursing work environment, compassion satisfaction, burnout, and secondary traumatic stress, *P*<0.05 indicated a statistically significant difference. Meanwhile, multiple linear regression analysis was used to examine the effects of participants' demographic and professional characteristics, traumatic stress, and nursing work environment on compassion satisfaction, burnout, and secondary traumatic stress.

# Results

**Demographic and professional characteristics of participants**

A total of 247 midwives completed the investigation, because of low quality (such as all options are consistent, like 1,1,1,1,1 or 5,5,5,5,5), 15 were excluded, and a valid response rate was 93.93%. Their mean age was 35.69 years (range: 22-54 years, SD=6.904). Of the 232 participants, 100% were female, 87.1% were married, and 82.3% held a bachelor’s degree. Most participants worked in a three-level hospital (66.8%) and had a supervisor nurse title (58.2%). More details are described in Table 1.

# The Professional Quality of Life, Nursing work environment and Traumatic stress

**measurements of midwives**

As shown in Table 2, based on Stamm, the means for the dimensions of professional quality of life show moderate compassion satisfaction, burnout, and low secondary traumatic stress. The majority of midwives had moderate compassion satisfaction (73.7%) and burnout (71.6%) levels, and low secondary traumatic stress (60.8%) levels, but no midwives reported high burnout and high secondary traumatic stress scores.

It was determined that the mean score for the nursing work environment was 121.12 (SD=21.106), and it was observed that the scores for the frequency of traumatic stress and the impact of traumatic stress were 7.18 (SD=4.077) and 24.17 (SD=9.256).

# Correlational results

The correlations between the main study variables are shown in Table 3. The nursing work environment score demonstrated a positive correlation with compassion satisfaction (r=0.610), while exhibiting negative correlations with burnout (r=-0.390) and secondary traumatic stress (r=-0.296). It showed midwives who reported a healthier nursing work environment reported higher compassion satisfaction level, lower burnout level, and lower secondary traumatic stress level. The frequency and impact of traumatic stress for midwives score showed a negative relationship with compassion satisfaction (r=-0.145, r=-0.383) but showed a positive relationship with burnout (r=0.254, r=0.452) and secondary traumatic stress (r=0.281, r=0.380).

# Factors associated with participants' compassion satisfaction, burnout and secondary traumatic stress

Nonparametric test results are shown in Table 1. Next, we used compassion satisfaction, burnout, and secondary traumatic stress as dependent variables, and selected variables with statistical significance (*P*<0.10) in univariate analysis and variables with statistical significance (*P*<0.05) in Spearman correlation analysis as independent variables for multiple linear regression analysis, Table 4 present the results. It was found that nursing work environment, the impact of traumatic stress, major shifts, health condition, and the frequency of traumatic stress had a statistically important effect on compassion satisfaction, and nursing work environment, the impact of traumatic stress and the frequency of traumatic stress accounted for 51.4%, 25.4% and 12.6% of the variance of compassion satisfaction. For

burnout, there were three variables (the impact of traumatic stress, nursing work environment, and health condition) that were statistically significant predictors, and nursing work environment and the impact of traumatic stress explained 19.9% and 39.0% of the variance in burnout. It was also found that the impact of traumatic stress, nursing work environment, and frequency of night shifts per month had a statistically significant effect on secondary traumatic stress, and nursing work environment and the impact of traumatic stress account for 17.2% and 33.8% of the variance of secondary traumatic stress.

# Discussion

Midwives are the key to ensuring the safety of mothers and infants and providing high-quality pregnancy health services. Paying attention to and improving midwives' compassion fatigue is conducive to safeguarding the rights and interests of midwives, promoting their physical and mental health, and improving nursing quality. In our study, compassion satisfaction and burnout level were moderate, and secondary traumatic stress level was low (according to the norms provided by Stamm). Compared with a survey of Italian midwives,^4^ the levels of compassion satisfaction, burnout, and secondary traumatic stress of Chinese midwives in this survey were similar. Compared with another survey among Iran midwives, this study revealed lower compassion satisfaction and secondary traumatic stress, and higher burnout.^37^ Compared with midwifery students in a university in northwest England,^38^ our study showed lower compassion satisfaction, higher burnout, and similar secondary traumatic stress. The scores of compassion satisfaction, burnout and secondary traumatic stress are also different due to different countries, sample size, culture, inclusion and exclusion criteria, and work environment.

The results of this study indicated that better health condition has a positive effect on

compassion satisfaction but a negative effect on burnout, which was consistent with other studies,^39,40^ and midwives who have poor health condition may have less energy, which affects their motivation to work, thus increase burnout. Moreover, better health condition has an important influence on the care nurses provide for their patients,^40^ health care workers with better health status experience higher levels of compassion satisfaction when helping others.^41^ This study also found that midwives working a day-night shift obtained lower compassion satisfaction than midwives working a day shift. An earlier study had also found

similar results.^42^ Midwives working in the day-night shift face the disorder of the biological clock and need to adapt to different lifestyles. Shift changes will affect their sleep and rest, resulting in fatigue and insufficient energy, reducing their ability to empathize and making them unable to get happiness and satisfaction from helping child-bearing mothers. Additionally, frequency of night shift can influence secondary traumatic stress. This is different from another study, which showed that frequency of night shift is related to compassion satisfaction and burnout but not secondary traumatic stress.^43^ Midwives working frequent night shifts may face more disruption in their sleep patterns and social life, and when facing trauma, they may be more stressed. Therefore, nursing managers should help manage the health of midwives and set up more reasonable schedules.

It is also found that traumatic stress is closely related to compassion satisfaction, burnout, and secondary traumatic stress. Most studies previously evaluated exposure to trauma and compassion fatigue.^4,26,44^ However, this is the first study to use a traumatic stress scale for midwives to assess its relationship to professional quality of life. The intimate relationship with child-bearing mothers is a double-edged sword,^44^ such emotional relationships can bring job satisfaction to midwives. However, it can lead to exposure to traumatic birth events. So, frequent witnessing and being affected by traumatic birth events may influence the job satisfaction of midwives, thus influencing the development of compassion satisfaction. The correlation analysis between the frequency and impact of traumatic stress and burnout corroborated a previous study,^34^ however, in the regression model, only the impact of traumatic stress was a predictor of burnout. It seemed that the frequency of traumatic stress did not reflect a stronger impact,^44^ perhaps it is the severity of the impact of the traumatic events that can significantly increase burnout. Burnout is characterized by elevated levels of emotional exhaustion, is not caused by trauma itself, but rather a result of chronic stress.^14^ The greater the impact of trauma, the more the emotional relationship with child-bearing mothers and a strong sense of responsibility make midwives remorse and guilt, which contribute to burnout.^34^ Moreover, the impact of traumatic stress is also an influence factor of secondary traumatic stress. Traumatic stress is frequently linked to directly experienced trauma, whereas secondary traumatic stress emerges from prolonged exposure to and empathic engagement with others' traumatic experiences. Consequently, it is comprehensible

that the immediate impacts of trauma are correlated with long-term effects. Hence, it is necessary to develop strategies for coping with trauma, such as trauma management and trauma-informed care education for midwives,^45^ in order to decrease the impact of compassion fatigue and improve compassion satisfaction.

It is worth noting that the nursing work environment was an important predictor of compassion satisfaction, burnout, and secondary traumatic stress. These results are consistent with some previous research findings,^25,46^ that the better nursing work environment, the better the midwives' compassion satisfaction and the worse the midwives' compassion fatigue. Career development can enhance professional level and comprehensive ability. Thus, a supportive work environment can improve the quality of the professional life of midwives. The doctor-and-nurse relationship is the provision of medical care for patients by nurses and doctors through open communication and coordination under the promise of equality, autonomy, and mutual respect,^47^ which is crucial to preventing compassion fatigue. Meaningful recognition may come in various forms, including affirmations by patients and their families, affirmations by other medical staff, satisfied pay, and value from work, which can be a powerful way to decease compassion fatigue and increase compassion satisfaction, and empowering tool to promote retention and reduce turnover.^48^ Sufficient human resources are about more than just the number of midwives available, it is also the staffing of human resources, which means a manageable workload. When the workload is unmanageable, people experience time pressure to accomplish their work as quickly as possible. However, time pressures may generate a feeling of overload, while also reducing the likelihood of reporting and taking time off, creating a vicious cycle and increasing the risk of compassion fatigue.^49^ Characteristics of effective leadership and management consists of encouragement, patient listening, and so on. The leaders, including the nurse managers and other managers, play an integral role in the midwives' enthusiasm and interaction and can be key to contributing to a healthy work environment.^48^ In addition, when gaining control at work, work will become more efficient, and a sense of fulfillment can be received in resolving certain conditions, which can be described as job satisfaction.^50^

In conclusion, despite moderate compassion satisfaction, burnout, and lower secondary

traumatic stress, it still suggests the importance of promoting self-care to enhance

compassion satisfaction and decrease compassion fatigue among midwives. Midwives may benefit from a better nursing work environment due to achieving stronger support when they face traumatic birth events and work in high-stress, high-risk situations. Thus, the human resource and nursing departments should work together to create a supportive working environment for midwives through trauma management and trauma-informed education to prevent and decrease compassion fatigue and promote midwives' physical and psychological health. In the future, research can be focused on larger sample sizes and large-scale studies, or longitudinal studies can be conducted.

# Limitations

Firstly, the cross-sectional design of the research limited the conclusions that can be drawn regarding compassion fatigue and compassion satisfaction. Secondly, there is potential for results bias due to using multiple self-reported questionnaires. Thirdly, the results are limited in generalizability since we used a convenience sample.

# Conclusions

This survey revealed that compassion satisfaction and burnout of Chinese midwives were at a moderate level, and secondary traumatic stress of Chinese midwives was at a low level. The nursing work environment and traumatic stress were important prediction factors for compassion satisfaction, burnout, and secondary traumatic stress. The above findings advise that nurse managers should pay attention to the physical and psychological health of midwives, develop targeted interventions, foster healthy work environment, and implement trauma management and trauma-informed care education to decrease midwives' compassion fatigue. The findings also provide valuable evidence for midwives' well-being, adding to limited knowledge in the field.

# Conflict of interest

The authors have no conflicts of interest.

# Supporting information

This research was supported by Henan Provincial Health Commission（Grant No: LHGJ20220551）.

# References

1. Dasan S, Gohil P, Cornelius V, Taylor C. Prevalence, causes and consequences of compassion satisfaction and compassion fatigue in emergency care: a mixed-methods study of UK NHS Consultants. Emerg Med J. 2015;32(8):588-594.

https://doi:10.1136/emermed-2014-203671 PMID: 25248545

2. Hassan TB. Averting compassion fatigue and burnout. Who should help us conquer our demons? Emerg Med J. 2015;32(8):586-587.

https://doi:10.1136/emermed-2015-204752 PMID: 25770123

3. Crowe L. Identifying the risk of compassion fatigue, improving compassion satisfaction and building resilience in emergency medicine. Emerg Med Australas. 2016;28(1):106-108. https://doi:10.1111/1742-6723.12535 PMID: 26774108
4. Guzzon A, Nones G, Camedda C, Longobucco Y. Exposure to Traumatic Events at Work, Post-Traumatic Symptoms, and Professional Quality of Life among Italian Midwives: A Cross-Sectional Study. Healthcare (Basel). 2024;12(4):415. https://doi:10.3390/healthcare12040415	PMID: 38391791
5. Leinweber J, Creedy DK, Rowe H, Gamble J. Responses to birth trauma and prevalence

of posttraumatic stress among Australian midwives. Women Birth. 2017;30(1):40-45. https://doi:10.1016/j.wombi.2016.06.006 PMID: 27425165

6. Hunter B, Berg M, Lundgren I, Olafsdottir OA, Kirkham M. Relationships: The hidden threads in the tapestry of maternity care. Midwifery. 2008;24(2):132-137. https://doi:10.1016/j.midw.2008.02.003	PMID: 18378051
7. Sheppard K. Compassion fatigue among registered nurses: connecting theory and research. Appl Nurs Res. 2015;28(1):57-59.

https://doi:10.1016/j.apnr.2014.10.007	PMID: 25434861

8. Jenkins B, Warren NA. Concept analysis: compassion fatigue and effects upon critical care nurses. Crit Care Nurs Q. 2012;35(4):388-395.

https://doi:10.1097/CNQ.0b013e318268fe09	PMID: 22948373

9. Houck D. Helping nurses cope with grief and compassion fatigue: an educational intervention. Clin J Oncol Nurs. 2014;18(4):454-458.

https://doi:10.1188/14.CJON.454-458	PMID: 25095300

10. Boyle DA. Countering compassion fatigue: a requisite nursing agenda. Online J Issues Nurs. 2011;16(1):2.

https://doi:10.3912/OJIN.Vol16No01Man02	PMID: 21800933

11. Stamm BH. The Concise ProQOL Manual, 2nd Ed;2010:13
12. Sullivan V, Hughes V, Wilson DR. Nursing Burnout and Its Impact on Health. Nurs Clin North Am. 2022;57(1):153-169.

https://doi:10.1016/j.cnur.2021.11.011 PMID: 35236605

13. Stamm BH. Helping the Helpers Helping the Helpers: Compassion Satisfaction and Compassion Fatigue in Self-Care, Management, and Policy of Suicide Prevention Hotlines;2012.
14. Sheen K, Spiby H, Slade P. Exposure to traumatic perinatal experiences and posttraumatic stress symptoms in midwives: prevalence and association with burnout. Int J Nurs Stud. 2015;52(2):578-587.

https://doi:10.1016/j.ijnurstu.2014.11.006	PMID: 25561076

15. Lake ET. Development of the practice environment scale of the Nursing Work Index. Res Nurs Health. 2002;25(3):176-188.

https://doi:10.1002/nur.10032	PMID: 12015780

16. Boudreau C, Rheaume A. Impact of the Work Environment on Nurse Outcomes: A Mediation Analysis. West J Nurs Res. 2024;46(3):210-218. https://doi:10.1177/01939459241230369	PMID: 38343035
17. Shin SH, Lee EH. Development and validation of a quality of healthy work environment instrument for shift nurses. BMC Nurs. 2024;23(1):37.

https://doi:10.1186/s12912-023-01672-4	PMID: 38212736

18. Fan SS, Zhou SQ, Ma J, An WH, Wang HH, Xiao T.The role of the nursing work environment, head nurse leadership and presenteeism in job embeddedness among new nurses: a cross-sectional multicentre study. BMC Nurs. 2024;23(1):159. https://doi:10.1186/s12912-024-01823-1	PMID: 38443951
19. Kelly LA, McHugh MD, Aiken LH. Nurse outcomes in Magnet(R) and non-magnet hospitals. J Nurs Adm. 2011;41(10):428-433. https://doi:10.1097/NNA.0b013e31822eddbc	PMID: 20431457
20. Manning J, Jones N. Improving Healthy Work Environments Through Specialty Nursing Professional Development. Journal of Radiology Nursing. 2021;40(3):241-245. https://doi:10.1016/j.jradnu.2021.05.006
21. Orton P, Gray DP. Burnout in NHS staff. Lancet. 2015;385(9980):1831. https://doi:10.1016/S0140-6736(15)60921-7	PMID: 25987150
22. Gribben JL, Kase SM, Waldman ED, Weintraub AS. A Cross-Sectional Analysis of Compassion Fatigue, Burnout, and Compassion Satisfaction in Pediatric Critical Care Physicians in the United States. Pediatr Crit Care Med. 2019;20(3):213-222. https://doi:10.1097/PCC.0000000000001803	PMID: 30418339
23. Sinclair S, Raffin-Bouchal S, Venturato L, Mijovic-Kondejewski J, Smith-MacDonald L. Compassion fatigue: A meta-narrative review of the healthcare literature. Int J Nurs Stud. 2017;69:9-24.

https://doi:10.1016/j.ijnurstu.2017.01.003	PMID: 28119163

24. Jensvold ML. A Preliminary Assessment of Compassion Fatigue in Chimpanzee Caregivers. Animals (Basel). 2022;12(24).3506

https://doi:10.3390/ani12243506 PMID: 36552426

25. Maillet S, Read EA. Areas of work-life, psychological capital and emotional intelligence on compassion fatigue and compassion satisfaction among nurses: A cross-sectional study. Nurs Open. 2024;11(2):e2098.

https://doi:10.1002/nop2.2098	PMID: 38391103

26. Qu LX, Gao JL, Liu L, Lun B, Chen DS. Compassion fatigue and compassion

satisfaction among Chinese midwives working in the delivery room: A cross-sectional survey. Midwifery. 2022;113:103427.

https://doi:10.1016/j.midw.2022.103427	PMID: 35853335

27. Hu L, Jiang M, Yang WQ. Investigation on the current situation of midwives human resources in China. Chin. J. Nurs. 2020;55(2):192-197. (in Chinese)

https://doi: 10.3761/j.issn.0254-1769.2020.02.006

28. Chen YH, Sun YJ. The Three-child Policy: Origin, Expected Effect and Policy Suggestions. Population and Society. 2021; 37(3):1-12. (in Chinese) https://doi:10.14132/j.2095-7963.2021.03.001
29. Ghaferi AA, Schwartz TA, Pawlik TM. STROBE Reporting Guidelines for Observational Studies. JAMA Surg. 2021;156(6):577-578. https://doi:10.1001/jamasurg.2021.0528	PMID: 33825815
30. Faul F, Erdfelder E, Buchner A, Lang AG. Statistical power analyses using G*Power

3.1: tests for correlation and regression analyses. Behav Res Methods. 2009;41(4):1149-1160. https://doi:10.3758/BRM.41.4.1149	PMID: 19897823

31. Ye ZH, Shao J. The development of Nursing Work Environment Scale and its reliability and validity. Chin. J. Nurs. 2016; 51(2):142-147. (in Chinese) https://doi:10.3761/j.issn.0254-1769.2016.02.002
32. Chen HY, Wang WH. Reliability and validity of the Chinese version of the Compassion Fatigue Scale. Chin. Nurs. Manag. 2013; 13(04):39-41. (in Chinese)

https://doi: 10.3969/j.issn.1672-1756.2013.04.012

33. Stamm BH. The ProQOL Manual: The Professional Quality of Life Scale: Compassion Satisfaction, Burnout and Compassion Fatigue/Secondary Trauma Scales;2005.
34. Kubota A, Horiuchi S. Traumatic stress experienced by Japanese midwives and its relation to burnout and work engagement. Jpn J Nurs Sci. 2023;20(1):e12505. https://doi:10.1111/jjns.12505	PMID: 35869599
35. Pu CS, Peng MY, Zhou CX, et al. Translation and validation of the Traumatic Stress Scale for Midwives. J.Nurs.Sci. 2023; 38(8):9-12.（in Chinese)

https://doi: 10.3870/j.issn.1001-4152.2023.08.009

36. Xiao Y, Liu F, Ran H et al. Resilience mediates the association between self-harm and suicidal ideation in Chinese left-behind children. BMC Public Health. 2021;21(1):2055. https://doi:10.1186/s12889-021-12153-1	PMID: 34753469
37. Hajiesmaello M, Hajian S, Riazi H, Majd HA, Yavarian R. How professional quality

of life is influenced by perceptions of empowerment in midwives. [British Journal of](https://www.scopus.com/sourceid/5000152303) [Midwifery](https://www.scopus.com/sourceid/5000152303).2022;30(2):80-90.

https://doi: 10.12968/bjom.2022.30.2.80

38. Beaumont E, Durkin M, Hollins MC, Carson J. Compassion for others, self-compassion, quality of life and mental well-being measures and their association with compassion fatigue and burnout in student midwives: A quantitative survey. Midwifery. 2016;34:239-244.

https://doi:10.1016/j.midw.2015.11.002	PMID: 26628352

39. Ryu IS, Shim J. The Influence of Burnout on Patient Safety Management Activities of Shift Nurses: The Mediating Effect of Compassion Satisfaction. Int J Environ Res Public Health. 2021;18(22):12210.

https://doi:10.3390/ijerph182212210	PMID: 34831966

40. Ryu IS, Shim JL. The Relationship between Compassion Satisfaction and Fatigue with Shift Nurses' Patient Safety-Related Activities. Iran J Public Health. 2022;51(12):2724-2732. https://doi:10.18502/ijph.v51i12.11463	PMID: 36742240
41. Jarrad R, Hammad S, Shawashi T, Mahmoud N. Compassion fatigue and substance use among nurses. Ann Gen Psychiatry. 2018;17:13.

https://doi:10.1186/s12991-018-0183-5	PMID: 29563960

42. Tahghighi M, Brown JA, Breen LJ, Kane R, Hegney D, Rees CS. A comparison of nurse shift workers' and non-shift workers' psychological functioning and resilience. J Adv

Nurs. 2019;75(11):2570-2578.

https://doi:10.1111/jan.14023	PMID: 30957259

43. Tang L, Wang F, Tang T. Exploring the relationship between family care, organizational support, and resilience on the professional quality of life among emergency nurses: A cross-sectional study. Int Emerg Nurs. 2024;72:101399. https://doi:10.1016/j.ienj.2023.101399	PMID: 38198948
44. Cohen R, Leykin D, Golan-Hadari D, Lahad M. Exposure to traumatic events at work, posttraumatic symptoms and professional quality of life among midwives. Midwifery. 2017;50:1-8.

https://doi:10.1016/j.midw.2017.03.009	PMID: 28347853

45. Long T, Aggar C, Grace S, Thomas T. Trauma informed care education for midwives: An integrative review. Midwifery. 2022;104:103197. https://doi:10.1016/j.midw.2021.103197	PMID: 34788724
46. Ni WW, Xia M, Jing MJ, Zhu SC, Li LM. The relationship between professional quality of life and work environment among ICU nurses in Chinese: a cross-sectional study. Front Public Health. 2023;11:1104853.

https://doi:10.3389/fpubh.2023.1104853 PMID: 37213646

47. Chen J, Zhang ZX, Xie H. Research progress on health-care partnerships. 2011; 46(11):1136-1139(in Chinese).

https://doi: 10.3761/j.issn.0254-1769.2011.11.044

48. Kelly L, Todd M. Compassion Fatigue and the Healthy Work Environment. AACN Adv Crit Care. 2017;28(4):351-358.

https://doi:10.4037/aacnacc2017283	PMID: 29212642

49. Figley CR. Treating Compassion Fatigue. New York: Brunner-Routledge; 2002.
50. Kong KYC, Ganapathy S. Are We in Control of Our Demons?: Understanding Compassion Satisfaction, Compassion Fatigue and Burnout in an Asian Pediatric Emergency Department in a Pandemic. Pediatr Emerg Care. 2022;38(3):e1058-e1062. https://doi:10.1097/PEC.0000000000002656	PMID: 35226631

**Table 1. Characteristics of the participants and univariate analyses of the demographic and professional factors associated with Compassion Satisfaction, Burnout, and Secondary Traumatic**

**Stress (N=232)**

| **Characteristics** | **Category** | ***n*(%)** | **CS** | | **BO** | | **STS** | |
| --- | --- | --- | --- | --- | --- | --- | --- | --- |
|  |  |  | ‾**x**±**s** | **Z/F**  **(*P Value*)** | ‾**x**±**s** | **Z/F**  （***P Value***） | ‾**x**±**s** | **Z/F**  （***P Value***） |
| **Ages(in years)** | ≦30 | 59（25.4） | 35.54±7.901 | 0.930  (0.628) | 25.10±4.521 | 1.204  （0.548） | 21.15±5.965 | 0.341  （0.843） |
|  | 31-40 | 120（51.7） | 34.61±7.995 |  | 25.61±4.133 |  | 21.56±5.203 |  |
|  | ≧41 | 53（22.8） | 36.08±6.782 |  | 24.94±4.601 |  | 21.75±5.547 |  |
| **Marital status** | Single | 24（10.3） | 35.79±7.616 | 2.461  (0.292) | 24.96±5.137 | 1.226  （0.542） | 20.33±5.903 | 2.240  （0.326） |
|  | Married | 202（87.1） | 34.98±7.730 |  | 25.41±4.253 |  | 21.70±5.448 |  |
|  | Divorced or windowed | 6（2.6） | 39.67±6.743 |  | 24.00±4.050 |  | 19.33±3.445 |  |
| **Education level** | Associate degree | 41（17.7） | 34.54±6.903 | -0.735  (0.462) | 24.76±3.992 | -0.885  （0.376） | 21.41±5.749 | -0.051  （0.959） |
|  | Bachelor’s degree | 191（82.3） | 35.32±7.875 |  | 25.45±4.404 |  | 21.52±5.416 |  |
| **Hospital level** | Three | 155（66.8） | 36.20±7.427 | -2.955  (0.003) | 24.94±4.180 | -1.826  （0.068） | 21.32±5.485 | -0.794  （0.427） |
|  | Two | 77（33.2） | 33.13±7.888 |  | 26.12±4.554 |  | 21.87±5.437 |  |
| **Professional title** | Primary nurse | 14（6.0） | 34.64±8.705 | 0.508  (0.917) | 24.43±4.926 | 1.329  （0.772） | 20.79±4.509 | 6.817  （0.078） |
|  | Senior nurse | 65（28.0） | 35.02±7.783 |  | 25.05±4.185 |  | 20.68±5.745 |  |
|  | Supervisor nurse | 135（58.2） | 35.16±7.704 |  | 25.46±4.244 |  | 21.59±5.426 |  |
|  | Deputy chief nurse | 18（7.8） | 36.39±7.114 |  | 26.06±5.207 |  | 24.33±4.715 |  |
| **Employment form** | Staffing at public | 71（30.6） | 35.69±6.863 | 2.226  (0.527) | 25.00±4.554 | 2.268  （0.519） | 21.38±5.131 | 0.615  （0.893） |
|  | Personal agency | 116（50.0） | 35.45±8.080 |  | 25.59±4.398 |  | 21.59±5.778 |  |
|  | Contractor | 42（18.1） | 33.83±8.076 |  | 24.98±3.879 |  | 21.62±5.277 |  |
|  | Temporary worker | 3（1.3） | 31.67±6.658 |  | 27.67±2.082 |  | 19.00±5.000 |  |
| **Duties** | No | 186（80.2） | 34.65±7.858 | -1.806  (0.071) | 25.23±4.344 | -0.565  （0.572） | 21.17±5.609 | -2.001  （0.045） |
|  | Head nurse | 46（19.8） | 37.33±6.700 |  | 25.72±4.319 |  | 22.83±4.654 |  |
| **Years as a midwife** | 1-5 | 187（80.6） | 34.58±7.831 | -2.227  (0.026) | 25.52±4.457 | -1.152  （0.249） | 21.42±5.458 | -0.433  （0.665） |
|  | ≧6 | 45（19.4） | 37.69±6.660 |  | 24.53±3.721 |  | 21.84±5.535 |  |
| **Major shifts** | Day shift | 90（38.8） | 37.61±6.347 | -3.737  (< 0.001) | 24.74±3.928 | -1.405  （0.160） | 21.58±4.972 | -0.437  （0.662） |
|  | Day-Night shift | 142（61.2） | 33.64±8.102 |  | 25.70±4.548 |  | 21.45±5.771 |  |
| **Frequency of night shift per month** | <5 | 85（36.6） | 35.40±7.593 | -0.189  (0.850) | 25.25±4.367 | -0.347  （0.728） | 22.53±4.755 | -2.426  （0.015） |
|  | ≧6 | 147（63.4） | 35.05±7.790 |  | 25.37±4.329 |  | 20.90±5.766 |  |
| **Personal monthly Income**  **(in Chinese Yuan)** | ≦5000 | 132（56.9） | 33.65±7.785 | 13.976  (0.001) | 25.33±4.533 | 0.663  （0.718） | 21.02±5.401 | 3.685  （0.158） |
|  | 5001-1000 | 96（41.4） | 37.15±7.123 |  | 25.26±4.135 |  | 22.00±5.483 |  |
|  | ≧10000 | 4（1.7） | 38.50±8.660 |  | 26.75±2.217 |  | 25.50±5.745 |  |
| **Weekly working time** | ≦50h | 154（66.4） | 36.00±7.514 | -2.095  (0.036) | 25.14±4.232 | -0.987  （0.323） | 21.71±5.195 | -1.032  （0.302） |
|  | >50h | 78（33.6） | 33.56±7.865 |  | 25.71±4.533 |  | 21.08±5.971 |  |
| **Number of deliveries per week on average** | 1-10 | 153（65.9） | 34.54±8.096 | -1.819  (0.069) | 25.30±4.440 | -0.092  （0.927） | 21.64±5.471 | -0.520  （0.603） |
|  | ≧11 | 79（34.1） | 36.42±6.759 |  | 25.38±4.149 |  | 21.23±5.475 |  |
| **Heath condition** | Very good | 59（25.4） | 39.15±5.539 | 24.325 | 23.46±4.606 | 26.892 | 20.80±6.122 | 2.331 |

|  | Good | 75（32.3） | 35.51±7.318 | （< 0.001） | 24.72±3.754 | （< 0.001） | 21.43±4.998 | （0.507） |
| --- | --- | --- | --- | --- | --- | --- | --- | --- |
|  | Neutral | 82（35.3） | 32.49±8.336 |  | 27.00±3.985 |  | 21.96±5.318 |  |
|  | Not good | 16（6.9） | 32.81±6.901 |  | 26.50±4.427 |  | 22.06±5.949 |  |
| **Sleep quality** | Very good | 23（9.9） | 40.65±5.540 | 15.685 (0.001） | 23.87±4.957 | 12.989  （0.005） | 19.35±5.951 | 8.422  （0.038） |
|  | Good | 50（21.6） | 36.16±7.675 |  | 24.00±3.974 |  | 20.40±5.660 |  |
|  | Neutral | 95（40.9） | 34.26±7.552 |  | 25.72±4.227 |  | 21.92±5.071 |  |
|  | Not good | 64（27.6） | 33.81±7.817 |  | 26.31±4.238 |  | 22.52±5.463 |  |

Abbreviations: BO, Burnout; CS, Compassion satisfaction; Secondary traumatic stress.

**Table 2. Midwives' Compassion Satisfaction, Burnout, Secondary Traumatic Stress, Nursing Work Environment Scale and Traumatic Stress Scale for Midwives**

|  | ‾**x**±**s** | **Range** | **High,n(%)** | **Moderate,n(%)** | **Low,n(%)** |
| --- | --- | --- | --- | --- | --- |
| **Professional Quality of Life** |  |  |  |  |  |
| CS | 35.18±7.703 | 12-49 | 47(20.3) | 171(73.7) | 14(6.0) |
| BO | 25.33±4.334 | 17-40 | 0 | 166(71.6) | 66(28.4) |
| STS | 21.50±5.464 | 10-35 | 0 | 91(39.2) | 141(60.8) |
| **NWES** | 121.12±21.106 | 52-156 |  |  |  |
| Career development | 23.80±4.806 | 5-30 |  |  |  |
| Leadership and management | 17.76±4.547 | 4-24 |  |  |  |
| Doctors and nurses relationship | 18.95±3.565 | 5-24 |  |  |  |
| Recognition atmosphere | 15.49±1.980 | 6-18 |  |  |  |
| Professional autonomy | 19.77±3.255 | 8-24 |  |  |  |
| Basic guarantees | 11.50±4.403 | 3-18 |  |  |  |
| Sufficient manpower | 13.85±3.056 | 3-18 |  |  |  |
| **TSSM** |  |  |  |  |  |
| Frequency | 7.18±4.077 | 0-28 |  |  |  |
| Impact | 24.17±9.256 | 0-45 |  |  |  |

Abbreviations: BO, Burnout; CS, Compassion satisfaction; NWES, Nursing Work Environment Scale; STS, Secondary traumatic stress; TSSM, Traumatic Stress Scale for Midwives.

**Table 3. Bivariate correlations (Spearman ’s) among Nursing Work Environment Scale, Traumatic Stress Scale for Midwives and Compassion Satisfaction, Burnout, Secondary Traumatic Stress**

|  | **CS** | **BO** | **STS** | **NWES** | **TSSM (frequency)** | **TSSM (impact)** |
| --- | --- | --- | --- | --- | --- | --- |
| **CS** | 1 |  |  | 0.610(＜0.001) | -0.145(0.028) | -0.383(＜0.001) |
| **BO** |  | 1 |  | -0.390(＜0.001) | 0.254(＜0.001) | 0.452(＜0.001) |
| **STS** |  |  | 1 | -0.296(＜0.001) | 0.281(＜0.001) | 0.380(＜0.001) |
| **NWES** | 0.610(＜0.001) | -0.390(＜0.001) | -0.296(＜0.001) | 1 |  |  |
| **TSSM (frequency)** | -0.145(0.028) | 0.254(＜0.001) | 0.281(＜0.001) |  | 1 |  |
| **TSSM (impact)** | -0.383(＜0.001) | 0.452(＜0.001) | 0.380(＜0.001) |  |  | 1 |

Abbreviations: BO, Burnout; CS, Compassion satisfaction; NWES, Nursing Work Environment Scale; STS, Secondary traumatic stress; TSSM, Traumatic Stress Scale for Midwives.

**Table 4. Multiple linear regression: predicting Compassion Satisfaction, Burnout, and Secondary Traumatic Stress**

| **Variables** | | | | ***B*** | ***SE*** | ***β*** | ***t*** | ***P Value*** | ***95% CI*** |
| --- | --- | --- | --- | --- | --- | --- | --- | --- | --- |
| **CS** | | | | 21.070 | 3.742 |  |  |  |  |
| NWES | | | | 0.187 | 0.020 | 0.514 | 9.182 | 0.000 | (0.147,0.228) |
| TSSM(impact) | | | | -0.211 | 0.046 | -0.254 | -4.590 | 0.000 | (-0.302,-0.120) |
| Major shifts | | | | -1.766 | 0.798 | -0.112 | -2.212 | 0.028 | (-3.339,-0.193) |
| Heath condition | | | | -1.056 | 0.437 | -0.125 | -2.416 | 0.016 | (-1.918,-0.195) |
| TSSM(frequency) | | | | 0.239 | 0.105 | 0.126 | 2.273 | 0.024 | (0.032,0.446) |
| R^2^=0.473 | Adjusted R^2^=0.462 | F=40.622 | *P*=0.000 |  |  |  |  |  |  |
| **BO** | | | | 23.924 | 2.056 |  |  |  |  |
| TSSM(impact) | | | | 0.183 | 0.027 | 0.390 | 6.667 | 0.000 | （0.129,0.237） |
| NWES | | | | -0.041 | 0.012 | -0.199 | -3.290 | 0.001 | （-0.065,-0.016） |
| Heath condition | | | | 0.865 | 0.271 | 0.182 | 3.191 | 0.002 | （0.331,1.400） |
| R^2^=0.331 | Adjusted R^2^=0.322 | F=37.565 | *P*=0.000 |  |  |  |  |  |  |
| **STS** | | | | 24.431 | 2.812 |  |  |  |  |
| TSSM(impact) | | | | 0.199 | 0.038 | 0.338 | 5.298 | 0.000 | (0.125,0.274) |
| NWES | | | | -0.044 | 0.016 | -0.172 | -2.697 | 0.008 | (-0.077,-0.012) |
| Frequency of night shift per month | | | | -1.449 | 0.670 | -0.128 | -2.164 | 0.031 | (-2.769,-0.130) |
| R^2^=0.207 | Adjusted R^2^=0.197 | F=19.894 | *P*=0.000 |  |  |  |  |  |  |

Abbreviations: Abbreviations: BO, Burnout; CS, Compassion satisfaction; NWES, Nursing Work Environment Scale; SE, standard error; STS,Secondary traumatic stress; TSSM, Traumatic Stress Scale for Midwives.
