# Supplementary material for: Correlations among the nursing work environment, traumatic stress, and professional quality of life in Chinese midwives: A cross-sectional study
Source: PLoS One. 2025 Oct 8;20(10):e0328686. doi: 10.1371/journal.pone.0328686 (PMC12507232; doi:10.1371/journal.pone.0328686)
Supplement: Attachment — Submitted filename: Response to Reviewers.doc [file pone.0328686.s003.doc]

**Point-by-point response to editors and reviewers**

Dear editors and reviewers:

On behalf of my co-authors, I would like to express our sincere gratitude for your valuable comments and suggestions regarding our manuscript. We have carefully considered your feedback and have made concerted efforts to incorporate your suggestions into the revised version. Below are your comments along with our corresponding responses:

**Response to editors**

Point 1:

Your manuscript, "*The correlations among the nursing work environment, traumatic stress, compassion fatigue, and compassion satisfaction among Chinese midwives: A cross-sectional study*”, has now been assessed.

Response 1: Thank you for your assessment of our manuscript titled "*The correlations among the nursing work environment, traumatic stress, compassion fatigue, and compassion satisfaction among Chinese midwives: A cross-sectional study.*" We greatly appreciate your feedback and the time you and the reviewers have dedicated to evaluating our work.

**Response to reviewer 1**

Reviewer #1:

All comments are included in the manuscript document.
The paper needs English editing.
The title addresses compassion fatigue and traumatic stress in the work environment; however, the whole paper rounds at CS,BO,and STS. Please clarify

Thank you for your insightful feedback.

1.The paper needs English editing.

Response:We accepted the comment. We used The American Journal Experts (AJE) for English editing.

2.The title addresses compassion fatigue and traumatic stress in the work environment; however, the whole paper rounds at CS,BO,and STS. Please clarify

Response:We accepted the comment, we have changed the title to “*The correlations among the nursing work environment, traumatic stress, compassion fatigue, and compassion satisfaction among Chinese midwives: A cross-sectional study*”.

Reviewer #2:

The study has good methodological quality and is relevant to the health field, contributing to the understanding of the relationship between work environment, traumatic stress and compassion fatigue among midwives. The research is of sufficient quality for publication, provided minor adjustments are made.
The title is clear and informative, reflecting well the main factors analyzed in the study. However, it could be more objective by reducing redundant information, such as repeating the word “work” and specifying the hospital context. Suggestion: The Correlation Between Nursing Work Environment, Traumatic Stress and Compassion Fatigue among Chinese Midwives: A Cross-Sectional Study.
The abstract presents a well-structured overview of the study, including context, objective, methodology, main results and conclusions. However, the contextualization could be more objective, and the results should emphasize more the practical importance of the correlations found, especially on how the work environment influences compassion fatigue.
The introduction sets out the problem well, highlighting the relevance of compassion fatigue among midwives and its impact on the health system. The literature review is well-founded, providing a solid foundation for the study. However, the justification could be more explicit, directly connecting the gap in the literature with the need for this research.
Methodologically, the study is well designed. However, the authors should clarify how many hospitals were initially eligible and how they arrived at the final sample of 59 institutions. Likewise, the estimated population of midwives and how the participants were approached should be better described. Were there any refusals to take part in the study? What was the response rate to the questionnaires? This information is essential for assessing the representativeness of the sample.
The results are presented objectively, with well-organized averages, standard deviations and percentages. The section correctly highlights how the work environment and traumatic stress impact compassion fatigue.
The discussion contextualizes the findings well in relation to the existing literature, explaining similarities and divergences with previous studies. The impact of the work environment on midwives' mental health is well explored, as is the importance of strategies to reduce compassion fatigue. However, it would be advisable to deepen the discussion on practical applications, suggesting concrete measures to mitigate the problem. In addition, the limitations section could be expanded, emphasizing how convenience sampling can influence the results.

Thank you for your comment.

1.The title is clear and informative, reflecting well the main factors analyzed in the study. However, it could be more objective by reducing redundant information, such as repeating the word “work” and specifying the hospital context. Suggestion: The Correlation Between Nursing Work Environment, Traumatic Stress and Compassion Fatigue among Chinese Midwives: A Cross-Sectional Study.
Response: Thank you for your suggestion!We have deleted some words “work”.

2.The abstract presents a well-structured overview of the study, including context, objective, methodology, main results and conclusions. However, the contextualization could be more objective, and the results should emphasize more the practical importance of the correlations found, especially on how the work environment influences compassion fatigue.

Response: We accepted the comment.We have revised the results in the abstract, particularly strengthening the impact of nursing work environment on compassion fatigue and compassion satisfaction.We appreciate this comment.

3.The introduction sets out the problem well, highlighting the relevance of compassion fatigue among midwives and its impact on the health system. The literature review is well-founded, providing a solid foundation for the study. However, the justification could be more explicit, directly connecting the gap in the literature with the need for this research.
Response: We accepted the comment. We have added gaps in previous research and the need and significance of this study.

4.Methodologically, the study is well designed. However, the authors should clarify how many hospitals were initially eligible and how they arrived at the final sample of 59 institutions. Likewise, the estimated population of midwives and how the participants were approached should be better described. Were there any refusals to take part in the study? What was the response rate to the questionnaires? This information is essential for assessing the representativeness of the sample.
Response:We accepted the comment.We have made detailed revisions to the methodology and made the process of distributing and collecting questionnaires clearer.

5.The discussion contextualizes the findings well in relation to the existing literature, explaining similarities and divergences with previous studies. The impact of the work environment on midwives' mental health is well explored, as is the importance of strategies to reduce compassion fatigue. However, it would be advisable to deepen the discussion on practical applications, suggesting concrete measures to mitigate the problem. In addition, the limitations section could be expanded, emphasizing how convenience sampling can influence the results.

Response: We accepted the comment. We have added discussions on practical applications, proposed specific measures to alleviate these problems, and also modified the limitations section, emphasizing how the convenience sampling can influence the results.We appreciate this comment.

In conclusion, we sincerely appreciate the time and effort you have dedicated to reviewing our manuscript. We hope that the revisions will make it acceptable for publication in “***PLOS ONE***”. Thank you once again for your valuable feedback!

Best regards,

Lixia Qu
