# Supplementary material for: Correlations among the nursing work environment, traumatic stress, and professional quality of life in Chinese midwives: A cross-sectional study
Source: PLoS One. 2025 Oct 8;20(10):e0328686. doi: 10.1371/journal.pone.0328686 (PMC12507232; doi:10.1371/journal.pone.0328686)
Supplement: Attachment — Submitted filename: Response_to_Reviewers_auresp_2.doc [file pone.0328686.s004.doc]

**Point-by-point response to editors and reviewers**

Dear editors and reviewers:

On behalf of my co-authors, I would like to express our sincere gratitude for your valuable comments and suggestions regarding our manuscript. We have carefully considered your feedback and have made concerted efforts to incorporate your suggestions into the revised version. Below are your comments along with our corresponding responses:

**Response to editors**

Thank you for submitting your manuscript to PLOS ONE. After careful consideration, we feel that it has merit but does not fully meet PLOS ONE’s publication criteria as it currently stands. Therefore, we invite you to submit a revised version of the manuscript that addresses the points raised during the review process.

Response: Thank you for your assessment of our manuscript titled "*The correlations among the nursing work environment, traumatic stress, compassion fatigue, and compassion satisfaction among Chinese midwives: A cross-sectional study.*" We greatly appreciate your feedback and the time you and the reviewers have dedicated to evaluating our work.

**Response to reviewer 3**

Thank you for the opportunity to review the manuscript titled “The correlations among the nursing work environment, traumatic stress, compassion fatigue, and compassion satisfaction among Chinese midwives: A cross-sectional study.” The study addresses an important gap in the literature by focusing on professional quality of life (ProQoL) in Chinese midwifery, a population often overlooked in global workforce studies. The methodology is appropriate, and the findings have significant implications for both practice and policy. However, several key issues need to be addressed to enhance the clarity, rigor, and impact of the manuscript.

Response: Thank you for your insightful feedback.

1.**Title & Abstract:** The current title includes repetitive phrasing (“among... among”). A more concise alternative is:

*"Correlations of Nursing Work Environment, Traumatic Stress, Compassion Fatigue, and Satisfaction in Chinese Midwives: A Cross-Sectional Study"*

Abstract (Results): Please clearly specify the direction of associations, such as:

“The nursing work environment was positively correlated with compassion satisfaction and negatively correlated with burnout and secondary traumatic stress…”

Response: We very much appreciate the time and effort you’ve put into your comments. We agree with the reviewer’s comment concerning this issue. We have taken into account your and Reviewer4’s and AJE’s opinions and changed the title to “*Correlations among the Nursing Work environment, Traumatic Stress, and Professional Quality of Life in Chinese midwives: A Cross-Sectional Study”* . Moreover, we have revised the (Abstract) results to make the associations direction clearer (lines 35-46).

2.Sampling and Participation: While you report a 26.49% participation rate from head nurses, it is unclear why 71 out of 268 agreed to distribute the survey. Please clarify if non-participating hospitals were systematically different (e.g., rural vs urban, secondary vs tertiary care).

-Response Rate & Bias: A 93.93% valid response rate is commendable, but the manuscript should address the potential for selection bias—for instance, whether more overburdened or burnt-out midwives were less likely to respond.

-Data Collection Tool: Briefly describe *Questionnaire Star*, e.g.:

“Questionnaire Star is a secure, widely used online platform for data collection in China, with features that limit duplicate responses.”

Response: Thank you for your valuable suggestion! We agree with your comment, in our data collection process, we have provided a clearer description (lines 139-142). Regarding “A 93.93% valid response rate is commendable, but the manuscript should address the potential for selection bias-for instance, whether more overburdened or burnt-out midwives were less likely to respond.” This is indeed a limitation of our research, and we have added it in the limitations section (line 393-395). Moreover, we have added the characteristics of Questionnaire Star to the Data Collection Tool as your suggestion (lines 144-145). We appreciate these comments

**3.Results**

-Demographics Table (Table 1): Ensure percentages for *all* categories are included. For instance, if 87.1% are married, what percentage are single, divorced, or widowed?

-Regression Reporting (Table 4): Specify whether standardized β coefficients were used. Clarify whether variance inflation factors (VIFs) were tested to rule out multicollinearity. Include adjusted R² values for all models—not only variance percentages.

Response: We acknowledge your comments very much, we accepted the comment. In table1, 87.1% are married, 10.3% are single, 2.6% are divorced or windowed (In the design questionnaire, these two are one option). Furthermore, in Regression Reporting (Table 4), we use standardized β coefficients, it is in the fourth column of Table 4 and we use abbreviations (*β*), the full name annotation is provided below the table. Regarding the question “whether variance inflation factors (VIFs) were tested”, we conducted the test according to your suggestion, and the VIF results are shown in Table 4, and an explanation of the VIF results was added in the final section of the results (the variance inflation factor (VIF) of all independent variables was below 5 (range: 1.094-1.351), indicating that there was no serious multicollinearity problem)(lines 264-266). We placed the result of adjusted R²values for all models in Table 4, and we revised it and move it at the bottom of Table 4, making it more clearer. We appreciate these comments.

**4. Discussion**

-Practical Applications: The discussion would benefit from specific examples of trauma-informed care education, such as:

“Structured debriefing sessions following traumatic deliveries, resilience training programs, and routine psychological support could be feasible interventions.”

-Cultural Context: Include commentary on how China's hierarchical and centralized healthcare system might reduce midwives' professional autonomy compared to Western nations, this is especially important when interpreting ProQoL findings.

-Policy Implications: Elaborate on the “three-child policy” and its impact. For example:

“The policy has increased the birth rate among older mothers, thereby increasing the complexity of deliveries and potentially elevating midwives’ exposure to traumatic events.”

Response: We thank for your constructive criticism, and time spent analyzing this

manuscript.We accepted the comment. Regarding “Practical Applications”, we have supplemented“Structured debriefing sessions following traumatic deliveries, resilience training programs, and routine psychological support could be feasible interventions.”in trauma-informed care education according to your suggestion (lines 335-336). Regarding “Cultural Context”, we have supplement how China's hierarchical and centralized healthcare system influence midwives ProQoL findings in DISCUSSION section according to your suggestion (lines 279-286). Regarding “Policy Implications”, we have supplement“three-child policy”and its impact in INTRODUCTION section according to your suggestion (lines 108-110). We appreciate these comments.

**5. Limitations**

Expand this section to include the following:

-Cross-sectional design: Acknowledge that the design prohibits inference of causality.

-Convenience sampling: Highlight the potential overrepresentation of tertiary hospitals (66.8% of the sample), which may not reflect midwifery conditions in smaller or rural hospitals.

-Self-reporting bias: Discuss the risk of social desirability bias, particularly underreporting of burnout or traumatic stress.

Response: Thank you for your suggestion! We have revised the limitations according to your suggestions in the section of Cross-sectional design, Convenience sampling and Self-reporting bias. These suggestions are very comprehensive (lines 385-395).

**6. References & Tables**

-Reference 49: Verify the PMID number and complete any missing data.

-Supporting Tables (1–4): These are labeled in the supplement but must be clearly cited in the main text (e.g., “See demographic details in Table S1”).

Response: We accepted the comment. We have verify the PMID number and complete any missing data. We added supporting tables, and labeled in the supplement and be clearly cited in the main text (lines 219-220).

7.**Critical Issues to Address**

7.1 In the Introduction, compassion fatigue is defined as consisting of BO + STS, but in the Abstract, CF, BO, and STS are treated separately. Please revise for conceptual consistency across the manuscript.

Response: We appreciate you pointing this out in your comment. We accepted the comment. We have revised the full text to all define professional quality of life and consist of compassion satisfaction, burnout, and secondary traumatic stress.

7.2 Regression Modeling Details: Indicate whether predictors were entered simultaneously or hierarchically in your regression models. Also, ensure all models report adjusted R², not just variance explained.

Response: We accepted the comment. In the regression model, it is entered by stepwise, which we have modified in the Data analysis (line 207.

7.3 Ethical Consent Clarification: The “Yes/No” question format is appropriate but specify whether this implied consent was explicitly approved by the ethics board, and whether written consent was waived.

Response: We acknowledge your comments very much, we accepted the comment. Regarding informed consent, we made additional modifications during the data collection process, described in detail as follows: the first question was set,“Do you agree to participate in this study?”After clicking“Yes”, you can start filling out the questionnaire (lines 147-148).

7.4 In the *Aim*, revise“nursing working environment”to“nursing work environment.”

Response: Thanks for your comment. We feel sorry for the error, we have revised it (line 20).

7.5 Ensure all abbreviations are defined at first use: BO = Burnout, STS = Secondary Traumatic Stress, CS = Compassion Satisfaction.

Response: We thank the reviewer for the kind comments. Abbreviations throughout the text have been corrected and harmonized.

7.6 Statistical Convention: The use of p < 0.10 for univariate tests is unconventional. Consider reverting to p < 0.05 or provide justification.

Response: Thank you for your suggestion! As you suggested, we have revised p < 0.10 into p < 0.05, and re-counted the multiple linear regressions (line 203, 250).

7.7 **References**
The reference list includes many regionally relevant studies but would benefit from the inclusion of additional international and contextually aligned literature to strengthen the manuscript’s theoretical foundations and policy discussion. For example, **Ayed et al. (2024)** examined the relationship between ICU nurses’ quality of life and their work environment, reinforcing the central research premise that institutional conditions critically influence caregiver well‑being ([*INQUIRY*](https://doi.org/10.1177/00469580241297974)). Similarly, **Batran et al. (2025)** explored the perceptions of neonatal nurses working in high‑acuity environments and found that elements of the work environment significantly shaped their professional quality of life—especially burnout and compassion satisfaction ([*PLOS ONE*](https://doi.org/10.1371/journal.pone.0322023)). In a related context, **Ejheisheh et al. (2025)** investigated the interplay between professional values and caring behaviors among ICU nurses in Palestine, offering both theoretical and empirical support for studying emotional resilience and value‑based care in high‑pressure healthcare settings ([BMC Nursing](https://bmcnurs.biomedcentral.com/articles/10.1186/s12912-025-02903-6)). The integration of these references would deepen the manuscript’s scholarly grounding and provide relevant international comparisons to contextualize the findings.

Response: We very much appreciate the time and effort you’ve put into your comments. We agree with your comment concerning this issue.We have read this literature carefully, it fits well with the context of our study, it really strengthens our theoretical foundations and policy discussions, and it is very useful for our article, which we have incorporated into our study (references 45, 44,19).

**Response to reviewer 4**

1.**Tittle:** the tittle gives multiple concepts making it difficult to differentiate dependent and independent variables I suggest “The correlations between nursing work environment, traumatic stress, and professional quality of life of Chinese midwives: A cross- sectional study”.

professional quality of life covers compassion fatigue, and compassion satisfaction. compassion fatigue however, includes burnout and secondary traumatic stress this need to rethink about having traumatic stress measured twice.

Response: We acknowledge your comments very much, we have taken into account your and Reviewer3's and AJE’S opinions and changed the title to “*Correlations among the Nursing Work environment, Traumatic Stress, and Professional Quality of Life in Chinese midwives: A Cross-Sectional Study*” . Regarding traumatic stress and secondary traumatic stress, we describe it in the third paragraph of our discussion, for example “In contrast to secondary traumatic stress, traumatic stress is less severe”. Your comment are valuable in improving the quality of our manuscript (lines 312-319).

2.**Abstract:** the background of the abstract focused mainly on compassion satisfaction and ignored other variables so please give more comprehensive background in the abstract. In results please revised this sentence as it requires clarification (The impact and frequency of traumatic stress accounted for 25.2% and 12.6%, respectively, of compassion satisfaction). Key words require revision to include main study variables. Suggestion *(workplace environment, compassion fatigue, compassion satisfaction, midwives, mainland China)*

Response: We are very grateful for your comments and thoughtful suggestions. As you suggested, we have revised the background of the abstract (focused on professional quality of life) (lines 12-18), results (described correlation analysis results and multiple regression results) (lines 35-46), and key words (lines 53-54).

3. **Introduction:** Also, in introduction you started with compassion fatigue, so you need to arrange your ideas along the manuscript starting with the tittle started with the work environment. At the end of your introduction please state the aim clearly.

Response: We are very grateful for your comments on the manuscript. We have reorganized the introductory section to begin with the context in work environment (lines 57-71) and stated the aim more clearly (lines 116-119.

4.**Methods:** please explain why you place the exclusion criteria, because it is not possible, all midwives should participate or attend continuing education sessions. For measuring sample size, what was the total number of midwives?

Please state the dependent and independent variables.

“With one head nurse from each hospital, and there were 268 head nurses in the group” what do you mean by the group? Please cite the reason where you excluded 15 responses.

The instruments were not arranged properly as the tittle and the remining of the paper.

What is the difference between secondary traumatic stress measure as part of the compassion fatigue and traumatic stress scale for midwives? Please clarify.

Response: Thanks for your valuable suggestions! I have re-described the exclusion criteria, in fact we mainly exclude midwives and who are not in post for various reasons and nursing students interning in the delivery room (lines 129-131).

Regarding “With one head nurse from each hospital, and there were 268 head nurses in the group what do you mean by the group? Please cite the reason where you excluded 15 responses”. we am sorry for our error, I meant Wechat group (line 139), we have revised it. Moreover, we explained the reason of excluding 15 responses in the end of Data collection process (lines 149-152).

Regarding “The instruments were not arranged properly as the tittle and the remining of the paper.” we have rearrange the instruction order as the tittle and the remining of the paper.

Regarding “What is the difference between secondary traumatic stress measure as part of the compassion fatigue and traumatic stress scale for midwives? Please clarify.”

We added elaboration in the discussion section to clarify the differences between them, for example “In contrast to secondary traumatic stress, traumatic stress is less severe” “Traumatic stress symptoms could serve as a prodromal marker for psychological conditions such as secondary traumatic stress ”(lines 312-319).

**5.Results:** you started with professional quality of life!! You need to organize your ideas at the same sequence along the paper.

Response: Thanks for your suggestions! We have reorganized the result order at the same sequence along the paper (lines 221-229).

**6.Discussion:** instead of writing compassion fatigue, and compassion satisfaction why you don’t write professional quality of life. Also, the introduction ideas need to be more organized.

Response: We acknowledge your comments very much, which are valuable in improving the quality of our manuscript. We've revised the paper in its entirety to professional quality of life.

**7.Conclusion:** please write your conclusion and avoid repeating the findings.

Response: We thank the reviewer for the kind comments. We modified the discussion section to avoid repeating the results (lines 398-399).

8. **Reference:** please ensure that all of the citations mentioned in the reference section

Response: We accepted the comment. We re-examined all the references.

9.the paper requires academic editing and English proofreading. Also, the author should organize his/her thoughts and arrange the study ideas along the paper to avoid confusion.

Response: We accepted the comment. We used AJE for English editing.

**Response to reviewer 5**

1. The introduction provides a comprehensive overview of compassion fatigue and its relevance to midwifery. However, it would benefit from 3 papers.

Response:We thank the reviewers for their constructive criticism, and time spent analyzing this manuscript. We have read these three papers carefully and they are so helpful that we have included them in the references to enhance the persuasive power of the paper (references 19.45.15).

2. The setting and recruitment procedures are well-described, but further explanation is needed regarding the generalizability of findings. Since the study used a convenience sample from one province (Henan), please discuss how representative this sample is of midwives in other Chinese provinces or nationally. Also, elaborating on the rationale for choosing Henan Province would strengthen the context.

Response: We thank the reviewer for the kind comments. We re-edited the recruitment procedure, and chose Henan Province also because of the convenience of sampling and one of the limitations of our study, this is described in Limitations (lines 389-391).

3. The manuscript is generally well-written, but there are sections with minor grammatical errors and awkward phrasing (e.g., “traumatic events, which are also traumatic for midwives”). A thorough language and grammar edit is recommended to improve clarity and professionalism.

Response: Thank you for your careful and detailed comments! We combed through the article in its entirety and used AJE for touch-ups and edits.

I appreciate the time and detail provided by each reviewer and have incorporated the

suggested changes into the manuscript to the best of my ability. The manuscript has

certainly benefited from these insightful revision suggestions. I hope that the changes I’ve made resolve all your concerns about the article. I’m more than happy to make any further changes that will improve the paper and facilitate successful publication. Thank you once again for your valuable feedback!

Best regards,

Lixia Qu
